# Supplementary material for: Crystal structure of a subtilisin-like autotransporter passenger domain reveals insights into its cytotoxic function
Source: Nat Commun. 2023 Mar 1;14:1163. doi: 10.1038/s41467-023-36719-2 (PMC9977779; doi:10.1038/s41467-023-36719-2)
Supplement: Supplementary file 1 — Supplementary Information [file 41467_2023_36719_MOESM1_ESM.pdf]

Supplementary information

Crystal structure of a subtilisin-like autotransporter passenger domain reveals insights into its cytotoxic function

Lilian Hor[1], Akila Pilapitiya[1], James A. McKenna[1], Santosh Panjikar[2,3], Marilyn A. Anderson[1], Mickaël Desvaux[4], Jason J. Paxman[1]*, Begoña Heras[1]*

[1]Department of Biochemistry and Chemistry, La Trobe Institute for Molecular Science, La Trobe University, Kingsbury Drive, Bundoora VIC 3086, Australia
[2]Australian Synchrotron, ANSTO, Clayton VIC 3168, Australia
[3]Department of Biochemistry and Molecular Biology, Monash University, Clayton VIC 3800, Australia
[4]Université Clermont Auvergne, INRAE, UMR454 MEDiS, 63000 Clermont-Ferrand, France

This PDF file contains:

Supplementary Figures 1 to 7
Supplementary Tables 1 to 5
Supplementary methods

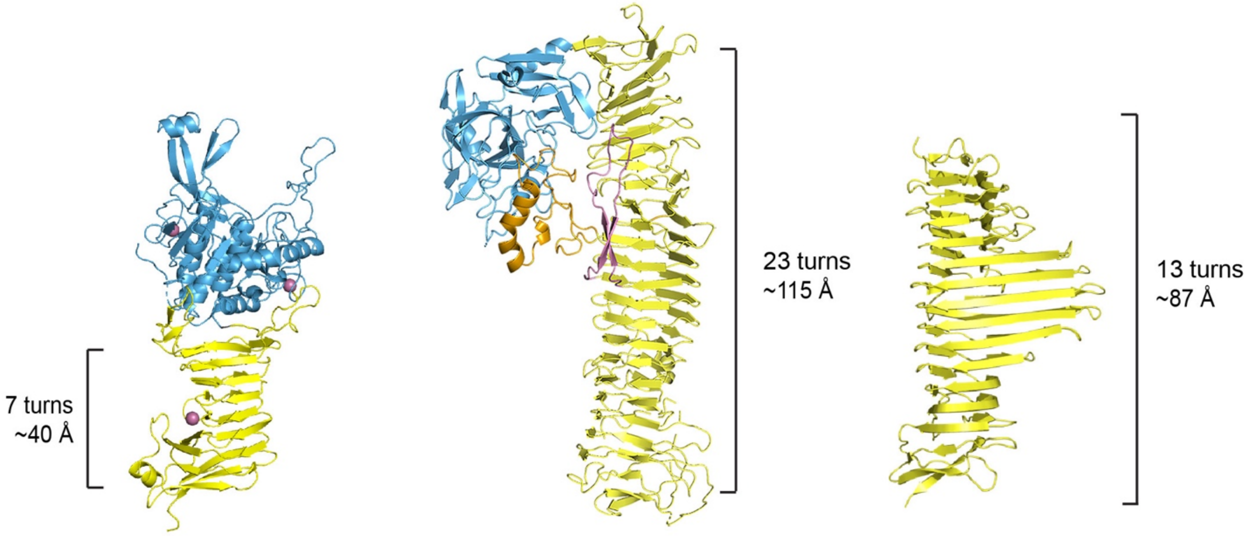

Supplementary Fig. 1. Comparison of the subtilase AT Ssp, SPATE Pet and adhesin AT UpaB. Crystal structure of Ssp (left), Pet (middle, PDB: 4OM9) and UpaB (right, PDB: 6BEA) showing the difference in the length of the β-helix, and the positioning of subdomains, in particular the protease domain, relative to the β-helix. β-helix is in yellow, protease domain in blue, other Pet subdomains in orange (d2) and pink (d4), and Ssp's calcium ions in pink spheres.

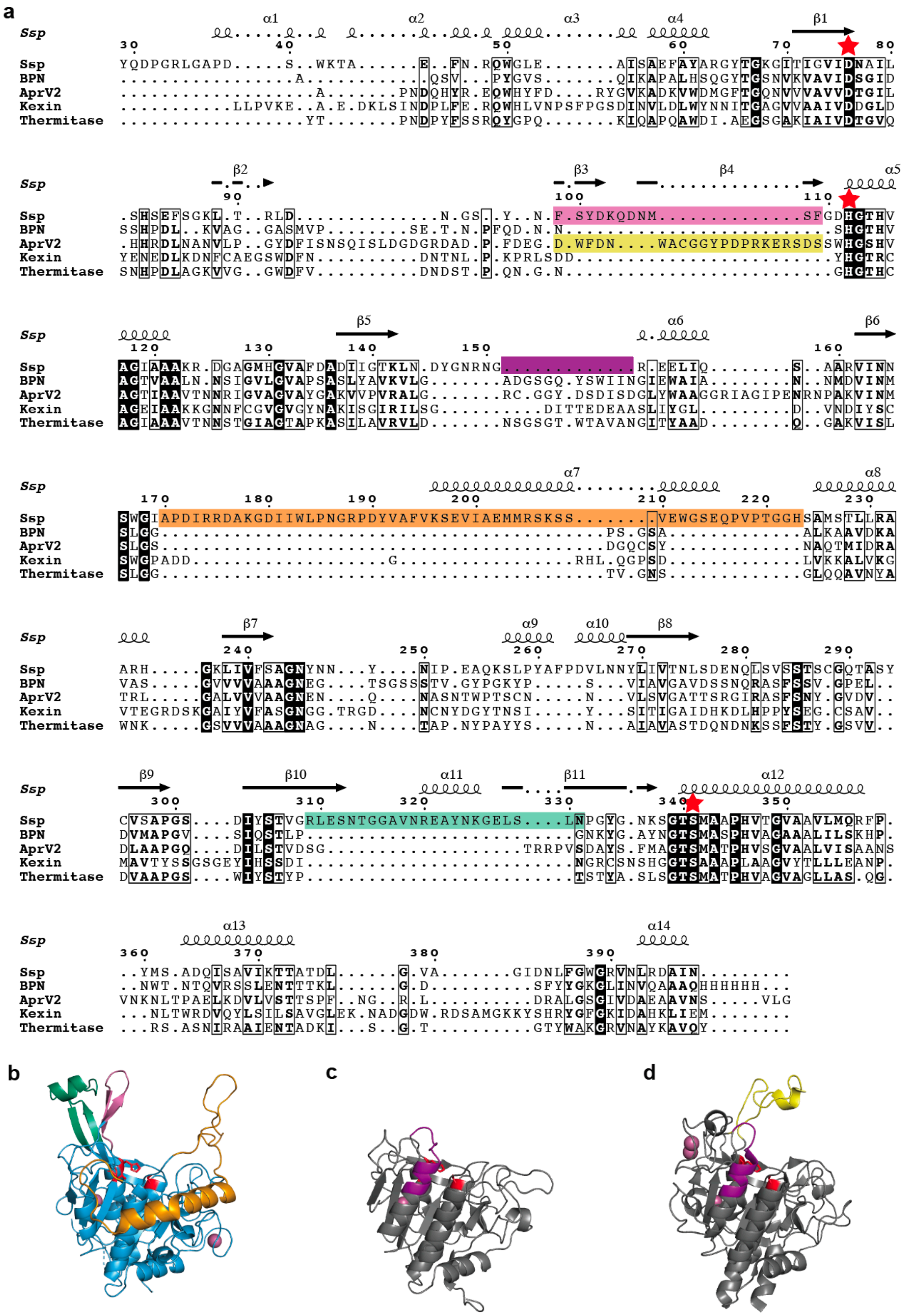

Supplementary Fig. 2. Structure based sequence alignment of the protease domain of Ssp and various subtilases. (a) Alignment with Ssp's active site extensions are highlighted, namely long β-hairpin extension (E3, green), short β-hairpin extension (E1, pink) and extended loop extension with connected α-helix (E2, orange). The Ssp active site deletion allowing for a wider substate binding site is shown in blue. AprV2 I2 insertion is highlighted in purple. Active site residues are denoted with red stars. BPN: subtilisin BPN'. Alignment was made with mTM-align [4] and visualised with ESPript 3.0 [5]. (b) Structure of Ssp protease domain. (c) Structure of subtilisin BPN' (PDB: 1LW6). (c) Structure of AprV2 (PDB: 3LPC). All structures are coloured as denoted in the alignment.

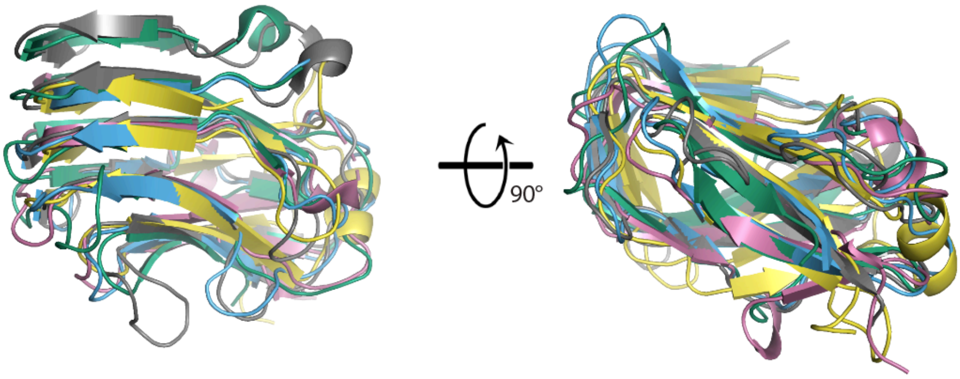

Supplementary Fig. 3. Overlay of Ssp's autochaperone (AC)-like domain with other autotransporter AC domains. Ssp (AC-like: G535–A642) in yellow, IcsA (PDB: 3ML3; AC: D606–D740) in grey, Hap (PDB: 3SYJ; AC: D830–P976) in green, P69 (PDB: 1DAB; AC: L444–P539) in pink, Ag43_EDL933 (PDB: 7KOH; AC: V453–E561) in blue.

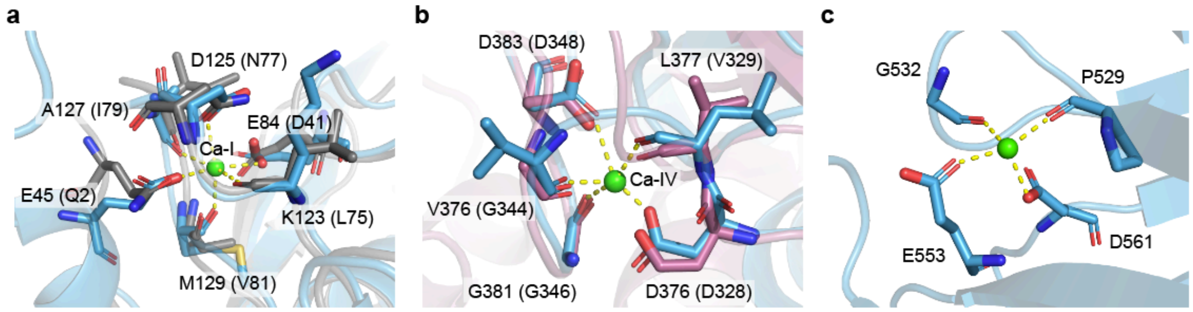

Supplementary Fig. 4. Coordination of calcium ions in Ssp. (a) Subtilase domain Ca-I (Dohnalek et al. nomenclature [6]). Overlay of the crystal structure of Ssp (blue) and subtilisin BPN' (grey, PDB: 1LW6). Residues which coordinate Ca-I are shown in sticks. Ssp residues are labelled with subtilisin BPN' residues in brackets. (b) Subtilase domain Ca-IV (Dohnalek et al. nomenclature [6]). Overlay of the crystal structure of Ssp (blue) and sedolisin (pink, PDB: 1GA1). Residues which coordnate Ca-IV are shown in sticks. Ssp residues are labelled with sedolisin residues in brackets. (c) β-helical domain calcium. Residues which coordinate the calcium ion are shown in sticks.

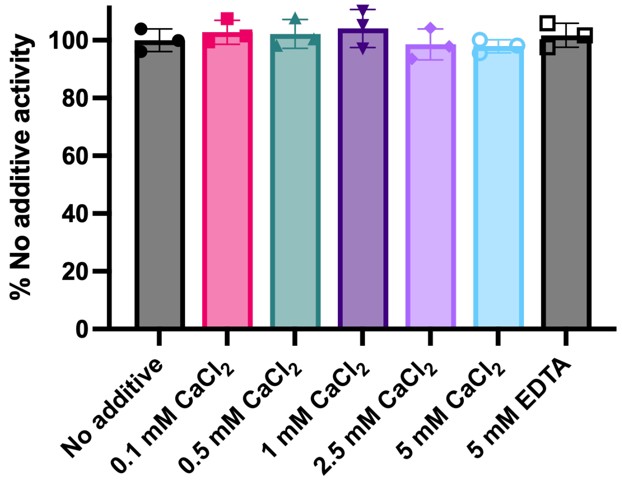

Supplementary Fig. 5. Activity of Ssp with various additives. Protease activity of the Ssp variants was measured using a fluorescent casein substrate in the presence of various concentrations of calcium and/or EDTA. Mean is plotted with error bars representing standard deviation of technical replicates (n=3). Data is representative of two independent experiments.

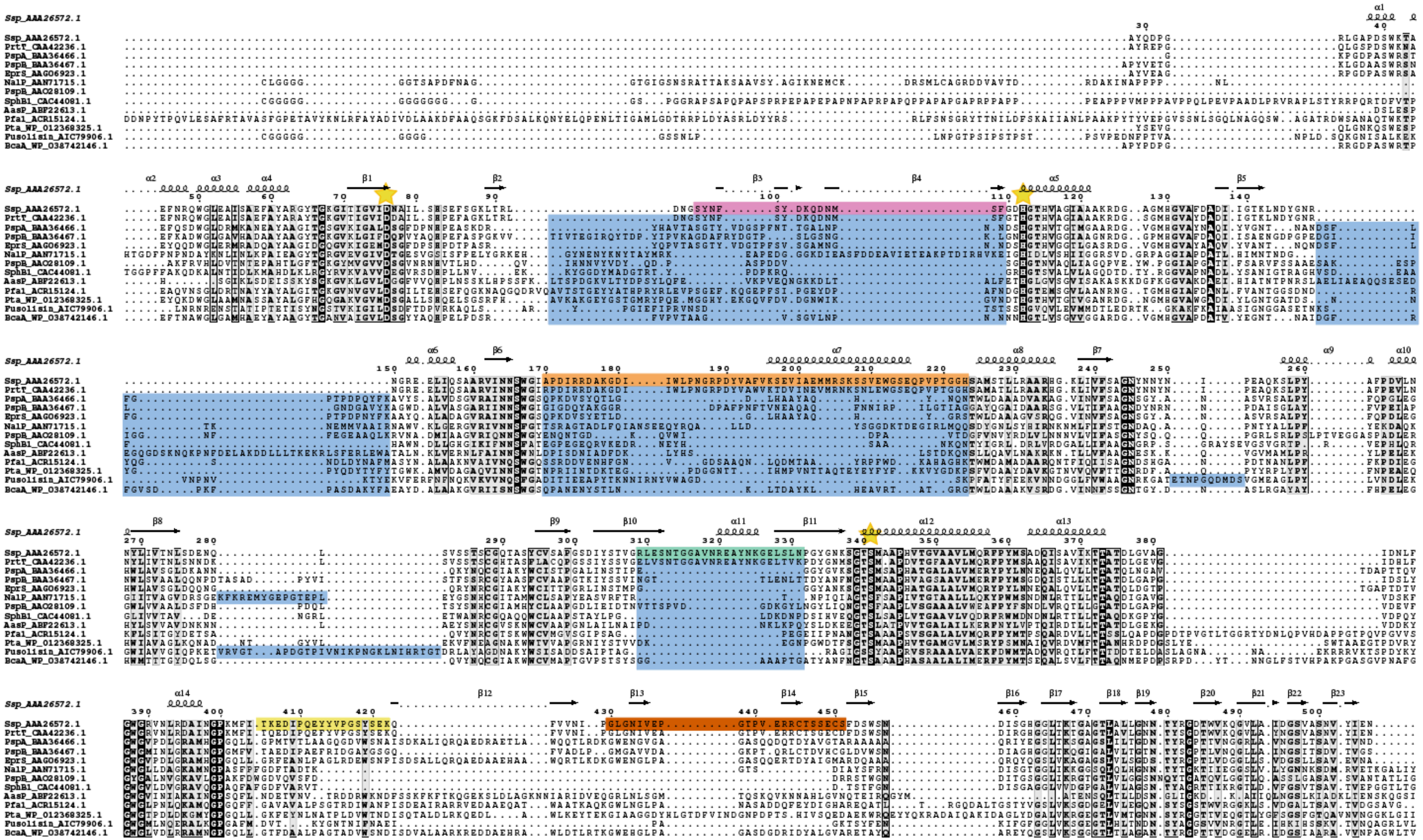

Ssp_AAA26572.1
PrtT_CAA42236.1
PspA_BAA36466.1
PspB_BAA36467.1
Eprs_AAG06923.1
NalP_AAN71715.1
PspB_AAC28109.1
SphB1_CAC44081.1
AasP_ABF22613.1
Pfal_ACR15124.1
Pta_WP_012368325.1
Fusolisin_AIC79906.1
BcaA_WP_038742146.1

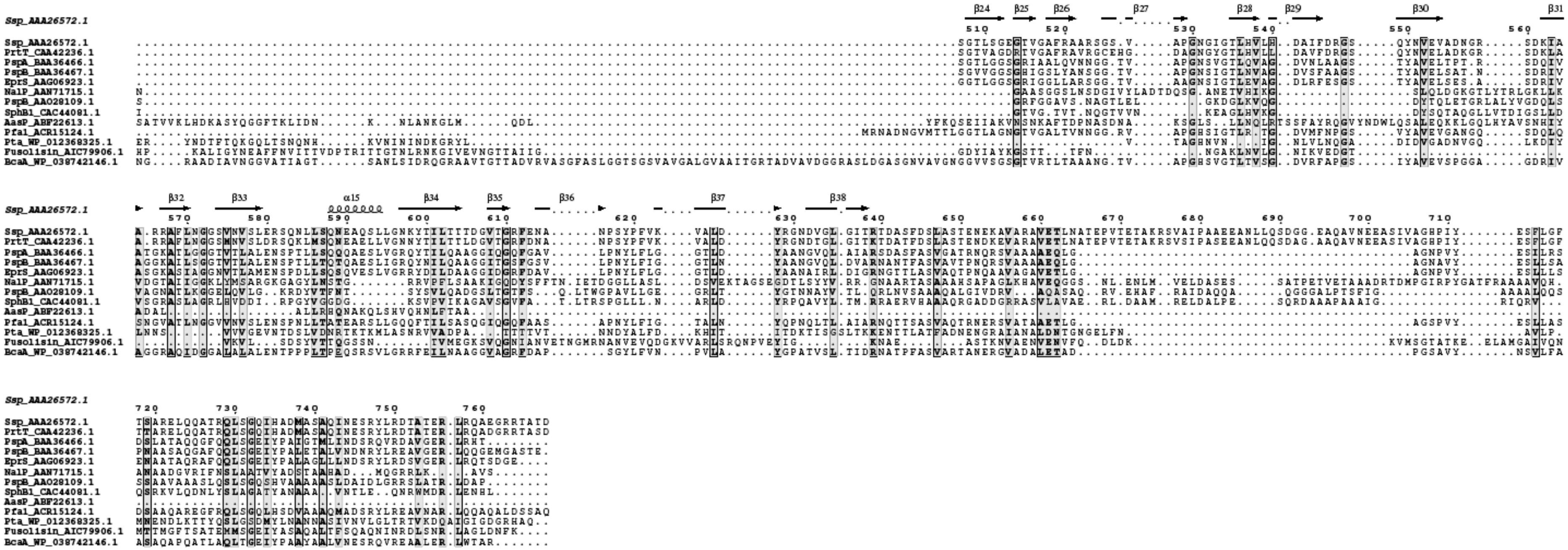

Supplementary Fig. 6. Sequence alignment of the predicted passengers of various subtilase ATs. The passengers of subtilase ATs were defined as the region downstream of the by predicted signal sequence as determined by SignalP 5.0 [7] and upstream of the translocator domain as determined by InterPro [8]. Ssp's unique active site extensions are highlighted, namely long β-hairpin extension (E3, green), short β-hairpin extension (E1, pink) and extended loop extension with connected α-helix (E2, orange). Potential subtilase AT active site extensions are highlighted in blue. Active site residues are denoted with yellow stars. Ssp's β-helix Loop 1 and Loop 2 are highlighted in yellow and rust, respectively. Alignment was made with Clustal Omega [9] and visualised with ESPript 3.0 [5].

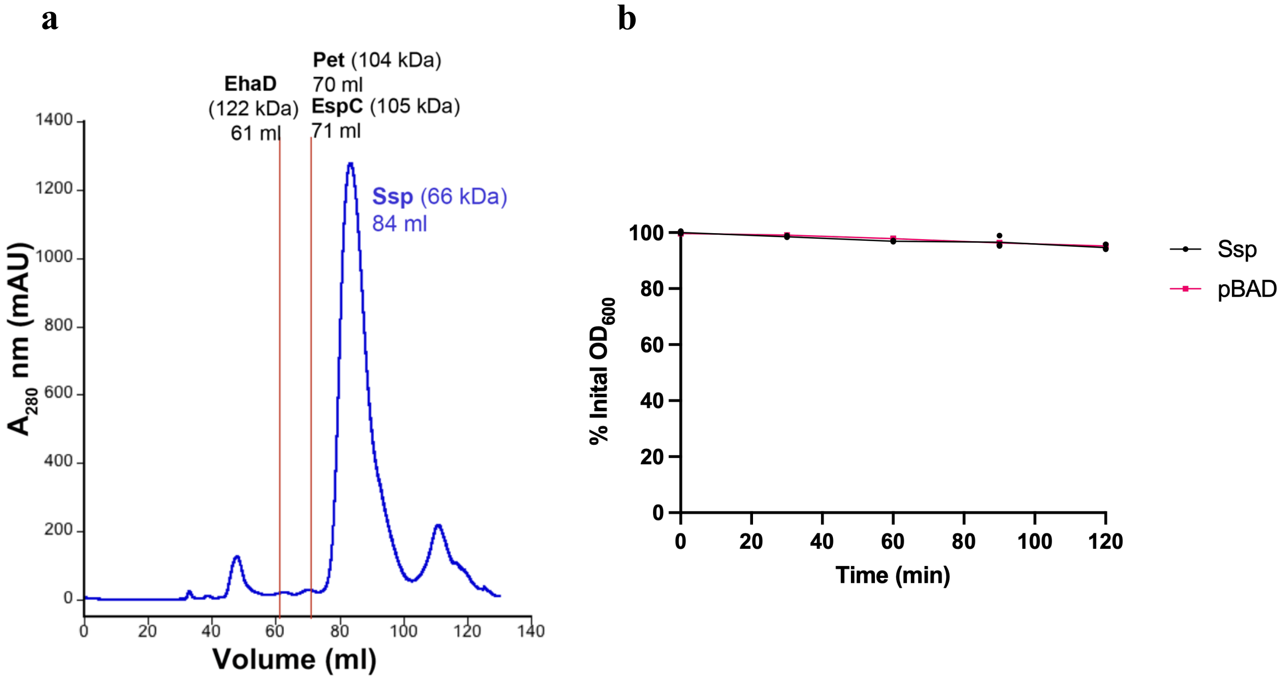

Supplementary Fig. 7 Size exclusion chromatography and Aggregation assay. (a) Size exclusion chromatography elution profile of Ssp (blue) (molecular weight 66 kDa and elution volume 84 ml). Vertical red lines indicate the elution profile of autotransporter proteins EhaD (molecular weight 122.2 kDa, elution volume 61 ml); Pet (molecular weight 104 kDa, elution volume 70 ml); EspC (molecular weight 105 kDa, elution volume 71 ml)). (b) Aggregation profile of *E. coli* Top10 pBADssp compared to pBAD vector only control. Cells were left to sediment for 2 h with OD_{600} measurements taken at 30 min intervals. Experiments were performed in triplicate with data points plotted with the means connected by a solid line. Note: Ssp is gradually cleaved and released by the actions of its own subtilase along with OmpT under these conditions.

Supplementary Table 1. List of all Ssp mutants generated.

Ssp mutant	Domain	Description	Protein expressed and stable
ΔL1	β-helical stalk	Deletion of residues 406–419 in Loop 1	No
ΔL2	β-helical stalk	Deletion of residues 433–452 in Loop 2	Yes
RAE	β-helical stalk	Mutation of RGD motif to RAE	Yes
S341A	Protease	Mutation of active site S341 to Ala	Yes
ΔE1	Protease	Deletion of residues 99–107 in E1 (short β-hairpin extension)	No
ΔE2	Protease	Deletion of residues 172–189 in E2 (extended loop, α-helix remains)	Yes
ΔE3+	Protease	Deletion of residues 308–332 in E3 (top half of long β-hairpin extension including α-helix)	No
ΔE3	Protease	Deletion of residues 316–325 in E3 (α-helix on top of long β-hairpin extension)	Yes
ΔE2/E3	Protease	Combination of ΔE2 and ΔE3 mutations	Yes

Supplementary Table 2. Ssp cleavage sequences. Primary sequence of known Ssp substrates [1]. Schechter and Berger nomenclature used with cleavage between P1 and P1'. Negatively charged residues are bold with asterisk.

Ssp cleavage site	P5	P4	P3	P2	P1	P1'
D645	**D***	A	S	F	**D***	S
E701	A	V	N	**E***	**E***	A
G716	**E***	S	F	L	G	F

Supplementary Table 3. Comparison of the structure of Ssp's autochaperone (AC)-like domain with reference autotransporter AC domains.

	IcsA AC (135 residues)	*Ag43_EDL933 AC (109 residues)*	*P69 AC (95 residues)*	*Hap AC (124 residues)*
Ssp AC-like (90 residues)	RMSD: 2.97 Å Over 64 Cα 10.9% identity	RMSD: 2.25 Å Over 60 Cα 18.3% identity	RMSD: 4.69 Å Over 38 Cα 5.6% identity	RMSD: 3.21 Å Over 59 Cα 8.5% identity
IcsA AC		RMSD: 2.62 Å Over 96 Cα 26.9% identity	RMSD: 4.12 Å Over 42 Cα 4.8% identity	RMSD: 1.63 Å Over 110 Cα 24.5% identity
Ag43_EDL933 AC			RMSD: 2.79 Over 67 Cα 16.4% identity	RMSD: 3.06 Over 75 Cα 24.0% identity
P69 AC				RMSD: 3.05 Over 79 Cα 30.4% identity

NB: RMSD was calculated using the secondary structure matching (SSM) superimpose tool [2] in Coot [3]. Aligned structures: Ssp (AC-like: G535–A642), IcsA (PDB: 3ML3; AC: D606–D740), Ag43_EDL933 (PDB: 7KOH; AC: V453–E561), P69 (PDB: 1DAB; AC: L444–P539) and Hap (PDB: 3SYJ; AC: D830–P976).

Supplementary Table 4. Thermal stability of Ssp. Apparent melting temperature (T_m^{app}) of Ssp as determined by UV/Vis spectroscopy.

Ssp	T_m^{app} (°C)	Difference (°C)
No additive	57.6 ± 0.1	-
+10 mM EDTA	56.7 ± 0.1	−0.9
+10 mM $CaCl_2$	61.5 ± 0.1	+3.9

Supplementary Table 5. Primers used to generate Ssp mutants.

Ssp mutant	Domain	Direction	Primer (5'–3')
ΔL1	β-helical stalk	Forward	ggCAGCGAAAAACAGTTTGTTGTTAATATTC
		Reverse	GGTAATGAACATTTTCGGACCATTAATCG
ΔL2	β-helical stalk	Forward	TTTGATAGCTGGTCAAATGATATTAGCGG
		Reverse	GCCCAGaCCAGGAATATTAACAAC
RAE*	β-helical stalk	Forward	CTATCGTGcTGAaACCTGGGTTAAACAG
		Reverse	CCAGGTtTCAgCACGATAGGTGTTATTAC
S341A*	Protease	Forward	AAAGtGGCACCgcaATGGCAGCACCGCATG
		Reverse	TGCCATtgcGGTGCCaCTTTTATTACCATAGCCAGGATTC
ΔE1	Protease	Forward	AGCTTTGGcGATCATGGCACC
		Reverse	GTTATAGCTACCATTATCCAGACGGG
ΔE2+	Protease	Forward	GATTATGTTGCCTTTGTTAAAAGCGAAG
		Reverse	CGGAGCAATACCCCAGCTATTG
ΔE2	Protease	Forward	GATTATGTTGCCTTTGTTAAAAGCGAAG
		Reverse	CGGAGCAATACCCCAGCTATTG
ΔE3	Protease	Forward	GGTGAACTGAGCCTGAATCCTG
		Reverse	ACCaGTATTGCTTTCCAGGCGAC

NB: Q5 Site-Directed Mutagenesis Kit (NEB) was used unless specified with asterisk (*) in which case the Stratagene QuikChange II method was employed. Lowercase letters denotes base pair mismatch.

Supplementary References

1 Shikata, S., Shimada, K., Ohnishi, Y., Horinouchi, S. & Beppu, T. Characterization of secretory intermediates of *Serratia marcescens* serine protease produced during Its extracellular secretion from *Escherichia coli* cells. *The Journal of Biochemistry* **114**, 723-731, doi:10.1093/oxfordjournals.jbchem.a124244 (1993).

2 Krissinel, E. & A Henrick, K. Secondary-structure matching (SSM), a new tool for fast protein structure alignment in three dimensions. *Acta Crystallographica Section D* **60**, 2256-2268, doi:doi:10.1107/S0907444904026460 (2004).

3 Emsley, P., Lohkamp, B., Scott, W. G. & Cowtan, K. Features and development of Coot. *Acta Crystallographica Section D* **66**, 486-501, doi:doi:10.1107/S0907444910007493 (2010).

4 Dong, R., Pan, S., Peng, Z., Zhang, Y. & Yang, J. mTM-align: a server for fast protein structure database search and multiple protein structure alignment. *Nucleic Acids Research* **46**, W380-W386, doi:10.1093/nar/gky430 (2018).

5 Robert, X. & Gouet, P. Deciphering key features in protein structures with the new ENDscript server. *Nucleic Acids Research* **42**, W320-W324, doi:10.1093/nar/gku316 (2014).

6 Dohnlek, J. *et al.* in *Understanding Enzymes: Function, Design, Engineering, and Analysis* (ed A. Svendsen) (Jenny Stanford Publishing, 2016).

7 Almagro Armenteros, J. J. *et al.* SignalP 5.0 improves signal peptide predictions using deep neural networks. *Nature Biotechnology* **37**, 420-423, doi:10.1038/s41587-019-0036-z (2019).

8 Blum, M. *et al.* The InterPro protein families and domains database: 20 years on. *Nucleic Acids Research* **49**, D344-D354, doi:10.1093/nar/gkaa977 (2020).

9 Sievers, F. *et al.* Fast, scalable generation of high-quality protein multiple sequence alignments using Clustal Omega. *Molecular Systems Biology* **7**, 539, doi:https://doi.org/10.1038/msb.2011.75 (2011).
