# Supplementary material for: Crystal structure of a subtilisin-like autotransporter passenger domain reveals insights into its cytotoxic function
Source: Nat Commun. 2023 Mar 1;14:1163. doi: 10.1038/s41467-023-36719-2 (PMC9977779; doi:10.1038/s41467-023-36719-2)
Supplement: Supplementary file 3 — Reporting Summary [file 41467_2023_36719_MOESM3_ESM.pdf]

nature portfolio

Corresponding author(s): Begona Heras, Jason Paxman

Last updated by author(s): Feb 4, 2023

Reporting Summary

Nature Portfolio wishes to improve the reproducibility of the work that we publish. This form provides structure for consistency and transparency in reporting. For further information on Nature Portfolio policies, see our Editorial Policies and the Editorial Policy Checklist.

Statistics

For all statistical analyses, confirm that the following items are present in the figure legend, table legend, main text, or Methods section.

n/a	Confirmed	
☐	☒	The exact sample size (*n*) for each experimental group/condition, given as a discrete number and unit of measurement
☐	☒	A statement on whether measurements were taken from distinct samples or whether the same sample was measured repeatedly
☒	☐	The statistical test(s) used AND whether they are one- or two-sided<br>*Only common tests should be described solely by name; describe more complex techniques in the Methods section.*
☐	☒	A description of all covariates tested
☒	☐	A description of any assumptions or corrections, such as tests of normality and adjustment for multiple comparisons
☐	☒	A full description of the statistical parameters including central tendency (e.g. means) or other basic estimates (e.g. regression coefficient) AND variation (e.g. standard deviation) or associated estimates of uncertainty (e.g. confidence intervals)
☒	☐	For null hypothesis testing, the test statistic (e.g. *F*, *t*, *r*) with confidence intervals, effect sizes, degrees of freedom and *P* value noted<br>*Give P values as exact values whenever suitable.*
☒	☐	For Bayesian analysis, information on the choice of priors and Markov chain Monte Carlo settings
☒	☐	For hierarchical and complex designs, identification of the appropriate level for tests and full reporting of outcomes
☒	☐	Estimates of effect sizes (e.g. Cohen's *d*, Pearson's *r*), indicating how they were calculated

Our web collection on statistics for biologists contains articles on many of the points above.

Software and code

Policy information about availability of computer code

Data collection	X-ray diffraction data were collected at the Australian Synchrotron on the MX2 Beam line using the QE Gui Eiger graphical interface (Aragao,D . et al. Journal of Synchrotron Radiation 25, 885-891 d(2018))
Data analysis	Protein structure determination by X-ray crystallography<br>-The X-ray diffraction data was indexed and integrated using XDS version Nov 1 2016 (Kabsch,W . Acta Crystallographica Section D 66, 133-144, (2010)) and scaled using AIMLESS version 0.7.4 (Evans PR,M urshudov GN. Acta Crystallographica Section D: Biological Crystallography. 2013; 69:1204-14)<br><br>- The anomalous data from SeMet crystals were analysed using BLEND version 0.6.23 (Foadi,J . et al. Acta Crystallographica Section D 69, 1617-1632 (2013)), whereby 2 datasets were merged using POINTLESS version 1.11.21 and AIMLESS version 0.7.4 (Evans PR,M urshudov GN. Acta Crystallographica Section D: Biological Crystallography. 2013; 69:1204-14).<br><br>-The structure was solved using a combination of SIRAS method and MRSAD phasing protocol in the Auto-Rickshaw platform version 2020/3 and FA values were calculated using the program SHELXC version 2006/2. (Panjikar,S . et al. Acta Crystallographica Section D 61,4 49-457 (2005); Sheldrick,G . Acta Crystallographica Section A 64,1 12-122 (2008); Selnium atoms were identified using SHELXD version 2006/2 Schneider,T . R. & Sheldrick,G . M. Acta Crystallographica Section D 58, 1772-1779 (2002)).<br><br>-Initial model generated using BUCCANEER version 1.6.5 (Cowtan,K. Acta Crystallographica Section D 62,1 002-1011 (2006)) with SAD refinement, and improved using the MRSAD phasing protocol of Auto-Rickshaw version 2020/3 (Panjikar, S., et al. Acta Crystallographica Section D 65, 1089-1097 (2009))

- The model was refined against the native dataset using REFMAC5 version 5.8.0253 (Murshudov, G. N. et al. Acta Crystallographica Section D 67, 355-367) and further built using COOT version 0.9.5((Emsley, P. & Cowtan, K. Acta Crystallogr D Biol Crystallogr 60, 2126-32 2004).

- The quality of the Ssp model was assessed by MolProbity (Davis, I.W. et al. Nucleic Acids Res 35, W375-83 2007)

- Protein structure figures were created with PyMOl version 2.5.3 (Delano, W.L. The PyMOl Molecular Graphics System, http://www.pymol.org/ . Delano Scientific, San Carlos, CA, USA. 2002

- Protein structural alignments DALI server (http://ekhidna.biocenter.helsinki.fi/dali_server/start)

For manuscripts utilizing custom algorithms or software that are central to the research but not yet described in published literature, software must be made available to editors and reviewers. We strongly encourage code deposition in a community repository (e.g. GitHub). See the Nature Portfolio guidelines for submitting code & software for further information.

Data

Policy information about availability of data

All manuscripts must include a data availability statement. This statement should provide the following information, where applicable:
- Accession codes, unique identifiers, or web links for publicly available datasets
- A description of any restrictions on data availability
- For clinical datasets or third party data, please ensure that the statement adheres to our policy

Data Availability
The crystallography, atomic coordinates, and structure factors reported in this paper have been deposited in the Protein Data Bank, www.pdb.org (PDB ID code 8E7F)

Other datasets used in this work are:

PDB: 5KE1 [http://doi.org/10.2210/ pdb5KE1/pdb]
PDB: 1LW6 [http://doi.org/10.2210/ pdb1LW6/pdb]
PDB: 1GA1 [http://doi.org/10.2210/pdb1GA1/pdb]
PDB: 3LPC [http://doi.org/10.2210/pdb3LPC/pdb]
PDB: 3EIF [http://doi.org/10.2210/pdb3EIF/pdb])
PDB: 3I6S [http://doi.org/10.2210/pdb3I6S/pdb])

Human research participants

Policy information about studies involving human research participants and Sex and Gender in Research.

Reporting on sex and gender	This study did not involve human research participants
Population characteristics	n.a.
Recruitment	n.a.
Ethics oversight	n.a.

Note that full information on the approval of the study protocol must also be provided in the manuscript.

Field-specific reporting

Please select the one below that is the best fit for your research. If you are not sure, read the appropriate sections before making your selection.

☒ Life sciences ☐ Behavioural & social sciences ☐ Ecological, evolutionary & environmental sciences

For a reference copy of the document with all sections, see nature.com/documents/nr-reporting-summary-flat.pdf

Life sciences study design

All studies must disclose on these points even when the disclosure is negative.

Sample size	Sample sizes were not predetermined based on statistical methods, but were chosen according to the standards in the field - In the Galleria studies, six larvae were randomly chosen per group as was performed in the following papers:<br>https://doi.org/10.1038/s42003-022-04197-9<br>https://doi.org/10.1021/acsomega.8b03578<br>https://doi.org/10.1080/21505594.2019.1659663

Data exclusions	No data was excluded from this manuscript
Replication	Reported results were replicated at least twice with experiments conducted on different days. All replicates generated similar results.
Randomization	Galleria larvae were chosen randomly from a pool of larvae in a petri dish. All larvae were sixth instar and of similar size.
Blinding	Investigators were not blinded. Blinding during analysis was not necessary because the results are quantitative and did not require subjective judgment or interpretation. Blinding is not typically used in the type of experiments.

Reporting for specific materials, systems and methods

We require information from authors about some types of materials, experimental systems and methods used in many studies. Here, indicate whether each material, system or method listed is relevant to your study. If you are not sure if a list item applies to your research, read the appropriate section before selecting a response.

Materials & experimental systems

n/a	Involved in the study	
☐	☒	Antibodies
☐	☒	Eukaryotic cell lines
☒	☐	Palaeontology and archaeology
☐	☒	Animals and other organisms
☒	☐	Clinical data
☒	☐	Dual use research of concern

Methods

n/a	Involved in the study	
☒	☐	ChIP-seq
☒	☐	Flow cytometry
☒	☐	MRI-based neuroimaging

Antibodies

Antibodies used	Rabbit polyclonal serum against Ssp was generated at the WEHI antibody facility, Melbourne, Australia.<br>Alexa Fluor Plus 647 conjugated goat anti-rabbit secondary antibody (Invitrogen, A32733)
Validation	Ssp antisera was initially generated using purified Ssp passenger domain and then validated at the WEHI antibody facility (Melbourne, Australia) by ELISA. This was confirmed by Western Blotting of Ssp-pBAD vs pBAD negative control E. coli supernatant samples, with detection using Antirabbit IgG/ HRP antibody was purchased from Promega (W401B).

Eukaryotic cell lines

Policy information about cell lines and Sex and Gender in Research

Cell line source(s)	HEp-2 cells were a gift from Dr Borg RMIT University Melbourne Australia
Authentication	Cell lines were not authenticated
Mycoplasma contamination	Cell lines were not tested for mycoplasma contamination
Commonly misidentified lines (See ICLAC register)	HEp-2 cells (HeLa) are used routinely in internalization assays in the autotransporter field. Thus, the cell line was reported as HEp-2 for consistency.

Animals and other research organisms

Policy information about studies involving animals; ARRIVE guidelines recommended for reporting animal research, and Sex and Gender in Research

Laboratory animals	Galleria mellonella larvae (sixth instar, gifted from the Laboratory of Professor Wieland Meyer, University of Sydney)
Wild animals	This study did not involve wild animals (Insects were laboratory reared)
Reporting on sex	Sex based analysis was not performed as it is very difficult to determine the sex G. mellonella larvae
Field-collected samples	This study did not involve samples collected from the field
Ethics oversight	Ethics approval is not required for this animal model

Note that full information on the approval of the study protocol must also be provided in the manuscript.
